# Community-based support for children who are next-of-kin for a parent experiencing illness or disability – a scoping review

**DOI:** 10.1186/s12913-021-07270-x

**Published:** 2021-11-19

**Authors:** Anne Kjersti Myhrene Steffenak, Agneta Anderzén-Carlsson, Elin Opheim, Tuva Sandsdalen

**Affiliations:** 1 Inland Norway University of Applied Sciences, Faculty of Health and Social Sciences, Elverum, Norway; 2 University Health Care Research Centre, Faculty of Medicine and Health, Örebro University, Örebro, Sweden

**Keywords:** Child, Children in community health, Intervention, Next-of-kin, Patient preference, Social services, Support

## Abstract

**Introduction:**

Children who are next-of-kin, for a parent who experience illness or disability, need support. In Norway, guidelines, routines and structured approaches in the community health services are lacking regarding involving children in the care of a parent and for services when supporting children as next-of-kin. Additionally, no existing international review has focused on support from community health and social services for children who are next-of-kin to a parent regardless of the specific illness or disability.

**Aims:**

This scoping review examined the current knowledge regarding the types of community health and social services support to children 0 to 17 years old living with a parent experiencing illness or disability. The review also identified children’s support preferences and needs.

**Methods:**

The scoping review involved five stages; identifying research question; identifying relevant articles; selecting articles; charting the data and finally, collating, summarizing and reporting the results.

**Results:**

Articles which included community health and social services interventions and children’s preferences or needs for support were included. The foci of interventions included preventive education, peer support, psychosocial support, and interventions focusing on family communication and recovery planning. Articles focusing on children’s preferences or need for support described their wish to be recognized as a next-of-kin, having someone to talk to and professional and peer support.

**Conclusion:**

The review highlighted the importance of children receiving support according to their preferences. It is important to elicit children’s voices, to ensure community health and social services are developed for and tailored to this population.

## Introduction

Children who are their parents’ next-of-kin include those who live with biological, adoptive, step- or foster parents experiencing illness or disability. We define disability in line with the UK equality Act (2010): including physical or mental disability that is a substantial and long-term and has adverse effect on a person’s ability to carry out normal day-to-day activities [1]. While the precise number is unknown, Maybery et al. estimate that, worldwide, one in five minors have a parent with a mental illness [2]. Adding children of parents with physical illness or disability would likely increase this estimate significantly [3, 4].

Children who are next-of-kin for a parent with an illness or disability work hard to understand what is happening and how to cope [5]. Children have a right to be involved in their parents’ conditions, have information and knowledge about their illness and have a voice in matters that affect their own situations in relation to this [6]. Nonetheless, children are often overlooked by services at a time when they need information about their parents’ state of health [7]; not knowing this information may increase their vulnerability [8]. They do not only need medical information (like what an illness is) but also learn how to adaptively cope with their parents’ illness [9].

More likely children who have been next-of-kin for a parent develop mental health problems such as anxiety and depression than children of healthy parents [10]. For instance, there appears to be a correlation between parents’ mental disorders and children’s behavioural difficulties and impaired psychosocial functioning [11]. Joseph et al. states, after summarizing research that 2–8% of children and young people can be defined as carers (varying from caring about to caring for a relative, irrespectively of diagnosis) and that this role may have a negative impact on their education, health, wellbeing, social opportunities and employment prospects [3]. Cuijpers et al. however, argue that it is hard to predict all known risk factors that develop mental disorders later in life among children living with a sick parent [12].

### Support for children from community health services

In community health care services, guidelines and routines regarding involving, identifying, giving information, advice and support to children as next-of-kin are lacking [13]. With community-based support we mean support provided by community health or social services, including care provided at community centres, primary health services and schools, as well as any of publicly or privately provided services intended to aid vulnerable persons or populations.

Guidance and long-term follow-up, it has been argued, should take place in the community where the children live [13]; however, there is often a lack of interaction between specialists and community health services around the follow-up of children who are next-of-kin. Structured interventions in clinical practice are a necessity, to ensure that children are approached in a manner that meets their needs and promotes their health and wellbeing. Positive coping strategies may increase young people’s level of mental health literacy [14]. The need for a child-friendly environment and the knowledge and experience of family-focused care is vital and should be prioritized. Depending on the level of a child’s involvement in the care of a next-of-kin their needs might vary, which should be taken into consideration when tailoring support [15]. According to Grove, et al., children need health care professionals that advocate for policies that support individual-, peer-, and family-focused programs that build on robust evaluation and research [9].

Globally, nurses work with children and their families, treating, supporting, teaching and following up on them in their everyday life, especially when they experience challenges. According to Foster et al. [15], nurses, such as the public health nurse, are in a key position to identify these children and address their needs. It is essential that children and their parents are able to know and trust that they can access support from health services when needed—as such, there must be a clear framework in place concerning the distribution of responsibility for this support [13]. In Norway, since public health nurses (PHNs) work in the community, in both health centres and school health services, they meet most of the children and their families living in the neighbourhoods [16, 17]. They may be best-positioned to identify, support and follow up on children who are the next-of-kin for an ill parent or a parent with a disability, and to provide them with support resources. To guide developing and implementing such work, a review of the scientific literature would offer a valuable synthesis of the current knowledge.

Many studies have examined support for children whose parents experience specific illness or disabilities, and literature reviews involving such articles have been conducted. However, these reviews largely centre around the children of parents with a specific illness [5, 11, 18]—most commonly a mental illness or cancer, or who have parents in palliative care [19, 20]. As such, the focus of these reviews is on the children’s experience of living with an ill parent, rather than on their support needs or experience with specific support interventions and services.

To our knowledge, no existing review has focused on community-based support, needs and preferences for support to children who are their parent’s next-of-kin, regardless of the specific illness or disability. Given the importance of support provision in the context in which these children live their daily life (i.e. at home), increased knowledge about their experiences with community-based support and their specific support needs and wants is fundamental. This insight informed by the children’s own voices and building on good evaluation and research is essential if community-based services are to be developed and tailored to this population.

### Aims

The previous scoping review examined the current knowledge of community-based support interventions for children 0 to 17 years of age living with a parent experiencing illness or disability, as well as these children’s needs and preferences for support from community health services when being a next-of-kin.

## Methods

This scoping review was conducted following the reporting guidelines provided by PRISMA [21] and the methodological framework of Arksey and O’Malley [22] further developed by Levac, Colquhoun and O´Brien [23]. This methodological framework includes the following stages: 1) identifying the research question; 2) identifying the relevant articles; 3) selecting the articles; 4) charting the data; 5) collating, summarizing and reporting the results; and 6) consulting (optional and not included in this review). In reporting, we adhere to the PRISMA guidelines for scoping reviews [21].

### Stage 1 identifying the research question

The study’s aim informed the eligibility criteria of this scoping review. The aim and eligibility criteria were in turn informed by the ‘interest, target group and outcome’ approach [23] which is often structured and referred to as PICO (P = patient/problem, I = intervention, C = comparison and O = outcome) [24]. In the present study, the target group was children who were their parents’ next-of-kin in the event of illness or disability (P); the focal point was on community-based support operated or organized by health professionals or social service staff (I); and the outcome of interest was twofold—interventions regarding support provided by community health services to children who were a parents’ next-of-kin, and the children’s support needs and preferences (O).

### Stage 2 identifying the relevant articles

#### Eligibility criteria

Articles were eligible for inclusion if they included 1) children who are their parents’ next-of-kin in the event of illness or disability, and 2) community-based support operated or organized by health professionals or social care staff and/or 3) children’s preferences, needs and outcomes with regard to support interventions from community health services. See Table 1.

**Table 1:** Eligibility criteria and PICO

Inclusion criteria	Exclusion criteria	PICO
Children from 0 to 17 years	Children from the age of 18 years	**P**: Children who are next-of-kin of parents in the event of illness: has a congenital or acquired illness or disability (physical or mental) including substance and gambling abuse
• Support to children of a parent who has a congenital or acquired illness or disability (physical or mental) including substance and gambling abuse	• Non-identifiable illness • Parents in forensic care • Parents in prison	
• Community based support, online support/interventions or equal, facilitated by health professionals or social care staff • Support offered to the individual child or as a family intervention or support offered to a parent with the aim of developing specific parental skills beneficial for an infant’s development. The studies should be included if they have a clearly described/determined child-foucused outcome. • Well-defined support	• Support given or organized by schools and non-health and social work staff • In-care support services • Hospital-based support • Foster-care, custody-related interventions • Family interventions if no specific child-focused outcome • Parent-focused interventions without child-focused outcome • Undefined or non-specific support • Support related to bereavement	**I:** Interventions regarding support for children **O**: Children´s preferences/needs and outcome of interventions regarding support for children from community health service
• Articles published from 2009 to 2019 English and Scandinavian languages	• Articles published outside the period 2009–2019 • Articles in other languages	**C:** Study design
• Empirical studies, theoretical papers (models) and reviews, if having a very similar focus on children • Quantitative studies: with outcome on child level • Qualitative studies: narratives from children describing what would have been valuable support (in a present or previous situation) and what was regarded as valuable support from the community health services; observations from professionals and parents with explicit focus on child outcomes • Mixed methods studies: see above (qualitative and quantitative study focus)	• Editorials, • Conference abstracts, • Dissertation abstracts, • Study protocols • Review articles, with other aim than ours • Outcomes which are not child focused	
• Articles using IMRAD structure	Studies where the IMRAD structure is missing, Non-articles, for example published dissertations	

No limitations were set concerning parental diagnosis, as children may need support regardless of their parents’ specific illness or disability. Joseph et al., in a narrative review, argues that it is not the parents’ diagnosis that determines what type of support the child needs, but rather how the child’s life is affected [3].

The limitation of the children’s age was up to 17 years, based on Article 1 of the Convention of the Children’s Rights [6]. As the present study targeted support provided or organized by community service professionals, articles focusing on support provided by non-professionals or hospital-based support were excluded.

Articles included in this review were theoretical papers (models) and empirical or review articles using the Introduction – Method – Results – and – Discussion (IMRAD) structure, published between 2009 and 2019, and written in English or a Scandinavian language, such as Swedish, Danish and Norwegian. The timeframe for the included articles was limited to 2009 to 2019, due to changes in the legislation and/or care policies concerning children in the authors’ countries of origin. As of 2010, the specialist health service in Norway began being required to have a child welfare advocate in each department [25]. This same year, children’s rights as next-of-kin were strengthened in Sweden, whose Medical Service Act (specifically, Chapter 5, §7) stated that the health service sector should recognize children of ill or impaired parents and ensure that their information and support needs are met [26]. The eligibility criteria are presented and elaborated in Table 1.

#### Search method

The search strategy was developed by the authors and the electronic database searches were conducted by an academic librarian (EO). Searches were performed from 3 December to 5 December 2019 in the following databases: CINAHL (EBSCO); Ovid MEDLINE (1946 to November 2019); OVID PsycINFO (1987 to November 2019); OVID Embase (1974 to November 2019); OVID AMED (1985 to November 2019); Cochrane Library (Wiley); Scopus (Elsevier); and Swemed. A combination of subject headings and text words for each element were used and adapted for the search strategy of each database. The full search strategy for CINAHL and Ovid MEDLINE is available from https://hdl.handle.net/11250/2688211. To enhance the coverage of relevant articles, the reference lists of identified review articles were reviewed for articles that had not been identified via the electronic search. Reference lists of included articles were also screened for relevant articles by the authors (AKMS, AAC and TS).

### Stage 3 selecting the articles—identifying relevant articles

**Fig. 1:**
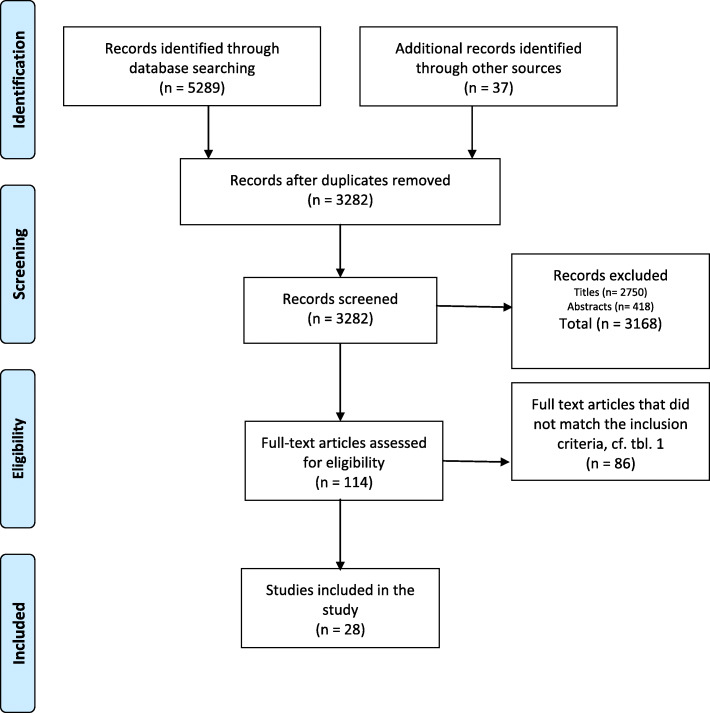
Flow Chart Identification and selection process of articles

A flow diagram (Fig. 1) shows the identification and selection process, as recommended by Tricco et al., (2018). The electronic database searches generated 5289 references and the manual searches of reference lists resulted in 37 references. Duplicates (*n* = 2031) were removed using EndNote X9 (Clarivate Analytics) and manual sorting, which led to a total of 3282 references. Titles and abstracts were screened in pairs by the authors (AAC, TMS, AKMS) for relevance related to the aim of the study and the eligibility criteria. This yielded a total of 114 articles to be assessed by the authors in full text. The articles were divided into three groups and the reviewers examined the articles in pairs. In each pair, the authors first independently read the articles and then carefully discussed all uncertainties or disagreements until agreement was reached. As a result, 87 articles were rejected, since they did not meet the eligibility criteria at this stage, leaving 28 articles to be included in this review.

### Stage 4 charting the data

Arksey and O’Malley [22] describe the charting process as multi-staged, involving extraction of information from individual articles. The researchers collectively developed the data charting form including which data to extract from the articles, as recommended by Levac et al. [23]. Data were extracted according to reference and country of origin, aim, study design and data collection methods, information about the participating children (age and gender), parental diagnosis, intervention/support given or preferences for support, and relevant outcome/findings. First, the data extraction was piloted for nine articles to ensure a mutual understanding of how to extract data relevant to the aim of this study; the interrater agreement for these was high. Then, data were extracted independently by the authors and discussed in pairs until consensus was reached. The results of the data charting process are shown in Table 2.

### Stage 5 collating, summarizing and reporting the results

The data were collated and summarized in three ways. First, to provide a structure for the identified articles, the articles were sorted alphabetically and given an individual number; the data extracted from each article were then collated and summarized in a data charting form (Table 2). Second, the data concerning the interventions were collated and summarized in three categories in a separate table (Table 3), according to type of intervention. Third, the process of collating and thematically summarizing the data regarding the children’s preferences for support was conducted (Levac et al., 2010), as presented in the text in the results section of this review article. The themes identified were based upon the findings described in the included articles. Similarities and differences in the findings were identified and grouped into themes, and the themes were then labelled and described. This process and the identification of the themes were discussed within the research group until consensus was reached. We did not use an inter-rater reliability process in the thematic analysis, since this is not specifically recommended in the method literature [21, 23].

## Results

### Overview of included articles

The included articles were published between 2009 and 2019, as shown in Table 2.

**Table 2:** Data charting form, data extraction from the included articles

			P: *Children who are next-of-kin of their parents in the event of illness*		I: *Interventions regarding support for children from community health service/preferences and expressed need for support*	O: Outcomes of *children’s preferences/needs and interventions regarding support*
Studies (***n*** = 28) First author, year, country	Aim	Study design/data collection	Participants	Parental diagnoses	Intervention/needs and preferences for support	Relevant outcome
**1 Bröning et al. (2019) Germany**	To examine a low-threshold psycho- education preventive intervention	Multicentre RCT, with pre- and post-tests 2 weeks prior, 1 week and 6 months after intervention. Questionnaires measuring: Stress, Coping, Health-related quality of life, Self-concept, Addiction-related knowledge	Intervention group: 130 children (68 males and 62 females), 6–14 yrs. Control group (fun-and play group): 88 children (46 males and 42 females), 6–14 yrs	Substance abuse	TRAMPOLINE (psycho-education preventive intervention)	Children from both groups reported reduced mental distress, reduced avoidance in coping with family stress, improved self-perceived autonomy, and a better parent–child relationship. Social isolation and addiction-related knowledge improved significantly more in the intervention group. No changes were observed in self-efficacy, physical stress symptoms, and in other health-related quality of life aspects.
**2 Davey et al. (2011) USA**	To explore how African American youth cope with the diagnosis and treatment of parental breast cancer, and to identify culturally sensitive ways to recruit and sustain participation of this vulnerable population in intervention programmes	Qualitative descriptive design. Focus groups	12 African American youth (3 males and 9 females), aged 11–18 yrs	Breast cancer	Preferences for support	A need for being included and appreciated by staff treating the parent was implicitly expressed. The youth described peer-support groups close to diagnosis and during treatment important, to talk, relax, have fun and get a break from cancer.
**3 Davey et al. (2013) USA**	To evaluate the effectiveness of a culturally adapted family intervention in improving family communication among African American parents coping with cancer and their school-age children. A secondary objective was to determine its impact on other symptoms of psychosocial distress (depression and anxiety). The third objective was to assess for acceptability and feasibility	Two-arm pre-intervention and post-intervention prospective design (2-year pilot). Data were collected at baseline and at end of treatment (10 weeks). Questionnaires measuring: Consumer satisfaction, Parent–adolescent relationship, General communication, Anxiety Depression. Open-ended narrative responses	19 children (8 males and 11 females), 10–18 yrs. from 12 families Intervention group: 7 African American families Control group (psychoeducation): 5 African American families	Various cancer diagnoses	Culturally adapted family intervention (CAFI)	Parents and children who completed the intervention reported significantly better communication with each other compared to the control group. Children and parents were more satisfied compared to those in the control group. No changes were noted in symptoms of anxiety or depression. Children especially valued the family session.
**4 Dunlop & Tsantefski (2018) Australia**	To explore child participants’ experience of EAT, with the aim of generating a greater understanding of how psychosocial outcomes occur, or do not occur, as a result of EAT	Qualitative descriptive design. Individual interviews	33 children (15 males and 18 females), 7–13 yrs. 28 children were interviewed	Substance abuse	Horse Club, an equine-assisted therapy programme	The children described emotional and physical safety and security with the horses and programme environment; feeling attachment and an opportunity for joy. Regarding personal and social development, they described finding new friends, mastering fears, increasing confidence and improving interpersonal behaviours.
**5 Foster et al. (2012) Australia**	To provide a framework for practice for nurses working with consumer parents in order to improve outcomes for parent, reduce burden of care for families, and provide preventive and supportive care for children	Theoretical paper		Mental illness	Family-focused care	Nurses could promote wellbeing in parent, child, and family by supporting the internal and external protective factors and reducing the risk factors
**6 Gladstone et al. (2014) Canada**	To examine children’s behaviours in and responses to the Children’s Group to understand whether they shared the goals of the programme, and how, or if, their needs were met	Qualitative descriptive design: A critical discourse analysis of the programme manual, Participant observation and informal interviewing during group interactions, Group interview with the children directly after the final session	7 children: 7–13 yrs	Mental Illness	A peer support group for children	Children were given explicit language to explain and talk about mental illness and their experiences and to articulate their needs and feelings. They developed an understanding about individual, yet common circumstances.
**7 Goodyear et al. (2009) Australia**	To extend the evidence base for the effectiveness of peer support programmes for children utilising outcome measures targeting self-esteem coping styles, connectedness and relationship problems, both within and outside the family unit	Observational study. Pre- and post-evaluation of a pilot intervention. Data were collected at baseline and 4 weeks after programme completion. Questionnaires measuring: Self-esteem, Coping skills, Connections and Relationship problems	69 children, 6–13 yrs. School holiday programme: 31 (7 males and 24 females) After-school programme: 38 (13 males and 38 females) 34 of the 69 children attended both programmes	Mental illness	CHAMPS (Children and Mentally ill Parents) School holiday and school peer support programmes	Post intervention, there were significant improvements in self-esteem, coping and connections within the family, and reductions in relationship problems for both programmes. The school holiday programme had the additional benefit of improving problem-focused coping. The impact on children’s wellbeing differed according to the intensity of the programme (consecutive days or weekly programme).
**8 Grove et al. (2015) Australia**	To examine the impact of the DVD on families and particularly how the intervention empowers children; 1. Children’s mental health knowledge and help-seeking behaviour pre- and post- exposure to the DVD; 2. Children’s perspectives regarding the DVD; 3. The ways in which the DVD had been employed by families and particularly children	Mixed methods design. Pre- and post-test. Questionnaire data were collected before watching the DVD and approximately 1 week after, focusing on: Mental illness-related knowledge, Help-Seeking, DVD evaluationz. Individual telephone interviews about the use of the DVD were conducted approximately 2–4 weeks after exposure to the DVD	29 children provided pre- and post-test questionnaire data (18 males and 11 females), 8–12 yrs. 18 children were interviewed (8 males and 10 females), 8–12 yrs	Depression and/or anxiety	Family-focus psycho-education DVD intervention, based on Beardslee’s family talk intervention	The children’s knowledge improved, and misconceptions were challenged. They rated themselves having been provided with some coping strategies, and ways of talking to someone in their family, besides their parents, about mental health issues. They preferred to talk to someone within the family, instead of someone outside the family. Most children preferred to watch the DVD together with a parent.
**9 Grové et al. (2016) Australia**	To present young people’s preferences for support and interventions	Mixed methods design. Study-specific questionnaire about what types of support young people want when a parent has a mental illness. Individual interviews	172 young people (all girls), 13–17 yrs. 6 of them participated in individual interviews, as well	Mental Illness	Preferences for support	94% of participants wanted support. They wanted information, to learn more about the illness, how to cope, knowledge that the mental illness is not their fault, as well as a place to relax. The interviews revealed that confidentiality and anonymity was an issue, as was the opportunity to talk to friends and to have support from school and healthcare professionals who are knowledgeable about the parental illness. DVD with information would be valuable on top of personal support.
**10 Landry-Dattée et al. (2016) France**	To evaluate the way in which the group responded to the various expectations of the children and to assess the impact of participation in the group on intra-family communication about cancer and on the children’s symptoms and the distress perceived and expressed by them.	A qualitative retrospective design (an evaluation of 12 yrs. of support groups for children and their parents). Individual interviews	19 children and young people (14 males and 5 females), 5–23 yrs.	Cancer	A parent–child group at the cancer centre (hospital and research centre)	The children expressed better understanding of the disease, reduction of symptoms such as distress and loneliness, and enjoyed meeting others in similar situations. They had reduced difficulty in speaking about cancer and increased knowledge about the hospital.
**11 Martinsen et al. (2019) Norway**	To investigate young next of kin’s need for information and involvement, to examine the ways they cope with situations involving coercion related to the treatment of their relative, and to identify ethical challenges	Qualitative descriptive design. Individual interviews	7 young next of kin, (3 males and 4 females), 14–22 yrs. 3 were a child of a person with mental health problems and 4 were siblings	Mental health problems	Preferences for support	The adolescents wanted information, and interaction with their sick relative at the hospital.
**12 Maybery et al. (2015) Australia**	To evaluate a family recovery planning model by examine the goals set by families with parents with mental illness (MI) and those families where a parent has a dual substance and mental illness (DD)	Observational study. Outcome variables were: the predetermined goals set by the children in the MI and DD groups after having completed three reviews of goals over a 12–18-month period	50 children from 10 mental illness families (10 males and 14 females) and from 10 dual illness families (16 males and 10 females)	Mental illness (MI) or dual substance and mental illness (DD)	Northern Kids Care—On Track Community Program (NKC-OTCP)	Important goals for children in both groups were related to education, interpersonal skills, mental health knowledge, family connectedness, lifestyle, child development and community, and social connectedness. The most important goal for both groups were education, as well as interpersonal skills for the MI group. During the programme period, the children in the MI group made good progress on goals related to acquiring mental health knowledge, but less progress on their other goals. Children in the DD group made good progress on all their most prominent goals and had greater progress toward goals than children whose parent had a MI.
**13 Maynard et al. (2013) Australia**	Identify what has been helpful for young people who have a parent diagnosed with cancer	Qualitative descriptive design. Individual telephone interviews with children whose parent had fallen ill with cancer during the last 5 yrs	15 children (6 males and 9 females), 14–18 yrs	Cancer	Preferences for support	Meeting the doctor who provided medical care to their parent was helpful for obtaining information and for humanizing the doctor. The children needed: time alone, time away and distraction from the cancer-affected environment and opportunities for emotional outlet. It was helpful that people at school were informed about the parent’s illness. Spending time with the ill parent and actively contributing to the family was considered important. It was helpful having extended social support including peers who had been through the same experience.
**14 McAndrew et al. (2012) England**	The study reports on the outcome of a participatory project aimed at better understanding the needs of young people caring for a parent	Participatory qualitative research design. After the event, the project team, together with the VOCAL group, established which of the issues raised were believed to be a priority to address with people attending the World Café	6 young carers who are members of VOCAL—the Forum for Young Carers in Salford, aged 13–17 yrs	Not defined	World Café event for young carers, the volunteer sector, health and social care practice, and education sector.	The issues that were believed to be most important to address for the volunteer sector, health and social care practice, and education were: Young carers need to be at the centre of discussions impacting on their lives. Organizations need to listen, engage, and have meaningful communication with young carers. Schools need to be flexible in order to meet the needs of young carers. Educators must listen to and recognize the experience of young carers. Young carers need practical and emotional support. They need a safe place to go where they can relax and where they will be listened to. There is a need for funding to continue and expand services.
**15 O’Neill et al. (2019) Ireland**	To examine how a group psychosocial intervention Children’s Lives Include Moments of Bravery (CLIMB®) helped young children to navigate parental cancer	Qualitative descriptive design. Focus groups, individual interviews and artwork (drawings and writing)	19 participants; 7 children (4 males and 3 females), 6–11 yrs. 7 parents	Cancer	A psychosocial child-focused group intervention (CLIMB®)	CLIMB® was evaluated as a positive experience for children through giving emotional support/navigating emotions, providing knowledge about cancer and related treatments, and by creating skills and space to talk about cancer and meet others in the same situation, which reduced feelings of isolation and alleviated anxiety
**16 Philips & Prezio (2017) USA**	To evaluate the outcomes of a community based psychosocial intervention targeted to children dealing with parental or primary caregiver cancer	Cross-sectional design. Secondary analysis of survey data, a multi-year sample of survey results. The data collection was completed within 6 months of the closure of the intervention. The questionnaires covered 9 topics related to the child: Sleep behaviours, eating habits, school performance, communication about illness, interacting with schoolmates, feeling secure at home, feeling anxious, relationship with ill family member and ability to separate from parent/caregiver	287 children (130 males and 157 females), 2- > 19 yrs., belonging to 156 families The primary respondent was the ill mother to these children	Cancer	Wonders & Worries (W&W) intervention	The amelioration of multiple children’s issues was reported, including: improved communication skills about illness, reduced anxiety, increased feeling of security at home, improved school performance, improved sleep behaviours, improved relationship with ill family member, eating habits and ability to separate from parent/caregiver. On all variables, some children’s issues remained stable and some worsened; here, most often reported was the difficulty to separate from parent/caregiver.
**17 Pihkala et al. (2012) Sweden**	To explore children’s experiences of the Family Intervention (FI) and what it meant for them to participate, and to explore parents’ perspectives on how the FI was for their children	Qualitative descriptive design. Qualitative interviews with children and parents the same year and the year after the family had participated in the intervention	14 children (7 males and 7 females), 6–17 yrs. and 14 parents from 9 families	Mental illness	Beardslee’s family intervention	The intervention led to increased knowledge and more open communication about parental illness and closeness to parents. The children also experienced a sense of relief from worry about the parent. Practical support at home arranged by professionals released the children from excessive responsibility and made it possible to spend more time with friends. There was a mutual understanding between parent and child about the effect of the FI. Two children experienced minimal benefits from the FI.
**18 Reupert et al. (2012)**	To identify issues when engaging children whose parents have a dual diagnosis in research, and present their needs and preferred support	Qualitative descriptive design. Qualitative interviews	12 children (6 males and 6 females), 8–15 yrs.	Dual diagnosis of mental illness and substance abuse disorders	Preferences for support	The children reported that their family needed support to become closer and more connected. The children needed someone to talk to about their feelings and support in managing their parent. Financial support was also needed.
**19 Riebschleger et al. (2009)**	To report early findings of a still-developing Youth Education and Support (YES) pilot intervention of multifamily group psychoeducation for youth with a parent with a psychiatric illness	Observational study with pre- and post-test design. Data were collected at the beginning of group one (pre-intervention) and at the end of the group six (post-intervention). Questionnaire measuring: Knowledge of psychiatric illness and recovery and Adolescent coping orientation to problem experiences	17 children (13 males and 4 females), 10–16 yrs.	Mental illness	Youth Education and Support (YES) pilot intervention	The children’s knowledge about psychiatric illness and rehabilitation/recovery increased. There was no significant improvement in coping after the programme.
**20 Semple & McCaugghan (2013) Ireland**	To explore the experience of families when a parent has cancer and the impact of a psychosocial intervention to support young children whose parent has cancer (CLIMB)	Qualitative descriptive design. Focus groups (separate for children and parents). Individual interviews with professionals delivering the psychosocial intervention. Data were collected 1 week after completionof the programme	7 children, (3 males and 4 females), 6–11 yrs) Parents, *n* = 6 Professionals, n = 2	Cancer	Children’s Lives Include Moments of Bravery (CLIMB)	Children sensed that something is wrong and wanted to know about parental illness. Children wanted to be involved; open communication rendered trust. CLIMB normalized the experience of parental cancer. It also improved children’s understanding of cancer, reduced misconceptions and equipped them with coping strategies. Communication about difficult emotions was eased. Children appreciated the peer support within CLIMB, meeting with children in a similar situation within a relaxed and fun environment. They felt less isolated and their experience was normalized. The intervention created a safe place, where they could forget about worries. The group in CLIMB should not be too small, to allow for dropouts during the intervention while remaining a group.
**21 Seng et.al. (2019) USA**	To describe the impact of parental migraine on adolescent children (aged 11–17) living at home with a parent with migraine	Cross-sectional observational study. Online survey to assess the impact of parental illness on children and a needs assessment on what helped to mitigate the impact of parental illness on children	40 parent–child dyads (10 male and 10 female children) with a mean age of 13.6 years	Migraine	Preferences for support	57.5% reported that services or interventions would be helpful. The most mentioned was having somebody to talk to about migraine (92.5%), having support from friends and family (90%), having ways to cope with anger (90%), and relying on parents for information about migraine (82.5%). The least mentioned was that it would help to talk to social services about any help provided (7.5%).
**22 Suchman et al. (2011) USA**	To investigate whether the benefits of MTP at posttreatment were sustained at the 6-week follow-up	A randomized pilot study. The control group took part in a parent education programme This was a 6-week follow-up study. Data for the purpose of this review were collected by means of observations of caregiving behaviour and child behaviour	47 mothers. The intervention group comprised 23 mothers, with a mean age of 31 years, whose child was about 19 months old The mean age of the 24 mothers in the control group was 29 years, and their child’s mean age was 17 months	Substance use	The Mothers and Toddlers Program (MTP)	The interaction between mother and child was moderately affected. No statistically significant effect was seen on child behaviour.
**23 Templeton et al. (2011) England**	To present qualitative data from children and young people who had attended three new family-focused services	Qualitative descriptive design. Individual interviews evaluating the intervention	23 young people (12 males and 11 females), aged 10–17 yrs)	Substance use	Moving Parents and Children Together (M-PACT), Base Camp, and Breaking the Cycle.	The young people were nervous before attending the service, not knowing who to meet, what to do and how to react. The young people benefitted through meeting other people in a similar situation (both children and adults) and having an opportunity to talk and share experiences, learn about addiction and understand and control their emotions. The programmes made the young people feel more positive. The worker involved in the programmes was an important figure. Their families became safer, healthier and more cohesive.
**24 Van Santvoort et al. (2014) The Netherlands**	To examine the effectiveness of Dutch support groups for children	Randomized Controlled Trial. Parents and children completed questionnaires at baseline and three and six months later Questionnaire for children measured: Child’s social support, cognitions, perceived competence and parent–child interaction Questionnaire for parents measured: Emotional and behavioural problems in the child.	Children from 254 families were randomly assigned to the intervention (*n* = 180) or a control group with three sessions of leisure activities (*n* = 74) 63.9% of the intervention group were female, vs 59.5% in the control group Mean age in intervention group was 10.37 yrs. vs 9.97 in the control group	Mental illness	Support groups for children aged 8–12 yrs	Children in the intervention group experienced a greater decrease in negative cognitions and sought more social support, immediately after participation and 3 months later, as compared to control group children. They also remained stable in their feelings of social acceptance (competence aspect) immediately after the intervention, whereas these feelings declined in control group children. The intervention and control groups both improved over time in terms of cognitions, competence, parent–child interaction and emotional and behavioural problem scores.
**25 Werner & Malterud (2017) Norway**	To explore informal adult support experienced by children with parental alcohol problems to understand how professionals can show recognition in a similar way	Qualitative descriptive design. Qualitative retrospective interviews about childhood and adolescence experiences	9 participants (3 males and 6 females), 25–54 yrs.	Alcohol abuse	Preferences for support	It was important to recognize the children’s needs and respond to these. These needs included social support, by providing a safe harbour and a sense of normality, for example being with friends and their families or a neighbour and escaping difficulties at home without having to talk about their problems. The need for practical support was also reported. Silent support not followed by an intervention or invitation to talk could lead to feeling of betrayal
**26 Woolderink et al. (2015) The Netherlands**	To gain knowledge about the expectations, experiences, and perspectives of participants and providers of the online Kopstoring course	Qualitative descriptive design. Individual interviews evaluating the intervention. No information given about the duration between intervention and interviews	13 children/young people (1 male and 12 females), 16- > 18 yrs. 4 providers of the intervention	Mental illness or addiction	Online preventive course (Kopstoring)	Participants experienced positive effects related to peer contact that led to feeling less alone, relieved, and less guilty. Learning about the illness or addiction of the parent gave insight into their parents’ behaviour. Learning how to cope with parents’ behaviour and problems led to acceptance and more family peace. All themes were perceived to be important for the course to be effective, especially the ‘rate your week’ part, which was a platform for questions and peer contact. Providers’ attitude and availability were appreciated. Online delivery was perceived to be ideal for participation in a safe and self-selected environment and the anonymity of the participant encouraged the participants to be more open. Barriers identified were related to: privacy in the home, technical problems, lack of time to discuss the homework assignments, too much focus on the younger participants, and complicated homework.
**27 Wong et al. (2017) UK**	To examine perceived social support among children of parents diagnosed with cancer, including positive and negative evaluations of various forms of social support	Qualitative descriptive design. Individual interviews	29 participants (9 males and 20 females), 18–38 yrs., who had been children (8–17 yrs) when the parent had cancer	Cancer	Preferences for support	The participants received and valued the following support: listening and understanding, encouragement and reassurance, tangible assistance, communication about cancer and treatment, and engaging in normal life experiences.
**28 Zeighami et al. (2018) Iran**	To explore the mental health needs of children of parents with mental illness	Qualitative descriptive design. Individual interviews	12 children of a parent with mental illness (4 males and 8 females), 17–26 yrs. Two healthy mothers, one daughter-in-law and four healthcare professionals	Mental illness	Preferences for support	The children’s needs changed in different phases of their parents’ illness In the pre-illness phase (early onset), they needed someone who understood and to whom they could talk about their problems. In the illness phase, they needed consolation and sympathy about the problems and the opportunity for rapid hospitalization. In the hospitalization phase, children did not like to be alone. In the borderline and the normal phases, children needed guidance on how to manage their newly-discharged parents and education about patient’s illness, care and legal issues.

Of the 28 included articles, all but one [27] were empirical articles describing the outcome of an intervention (Table 3) or children’s preferences or expressed need for support when being a next-of-kin.

The parental illness in the articles varied. A majority focused on mental illness of some kind (*n* = 10), cancer (*n* = 8), alcohol/substance abuse/use (*n* = 5) or mixed diagnoses of mental illness and alcohol/substance abuse/use (*n* = 3). In one article, the parents experienced migraine, and in another article, the diagnoses were not specified. None of the included articles focused on parental disability. The empirical articles were conducted in 11 different countries, on 4 continents: Europe (*n* = 11), North America (*n* = 8), Oceania (*n* = 7) and Asia (*n* = 1). The theoretical paper was written in Australia [27].

Eleven articles focused on empirical outcomes of interventions primary focusing on the child, one focused on support offered to mothers of children up to 36 months [28] and six articles described outcomes from family interventions (Table 3). The remaining ten articles described the results from articles focusing on children’s preferences or expressed need for support. The study design in the articles varied; most of the articles employed a qualitative descriptive design (*n* = 16), based on individual interviews (*n* = 8) or focus groups (*n* = 1). In three articles, it is not specified whether the interviews were individual or focus groups. Finally, multiple qualitative data collection methods were used in four articles.

**Table 3:** Support and interventions

Type of intervention	Study	Content of the intervention
**To the children**		
Group programme (TRAMPOLINE)	1 Bröning et al. (2012)	This intervention included nine group modules for children, containing specific addiction-related themes, provided on a weekly basis and conducted by trained social workers. The aim of the intervention was to help children cope with stress and develop a positive concept of self.
Equine-assisted therapy (EAT)	4 Dunlop & Tsantefski (2018)	This intervention included an equine-assisted therapy programme for children, consisting of two-hour sessions per week over 9 weeks. It was led by a trained Equine-Assisted Growth Practitioner, as part of an alcohol and other drug treatment service for children whose parent has a substance abuse.
Peer-support group	6 Gladstone et al. (2014)	An eight-week, psycho-educational and peer-support programme for school-aged children. Children were expected to learn mental illness information because ‘knowledge is power’, and to express difficult feelings about being a child of a mentally ill parent. The group was led by an experienced facilitator and one who was in training.
School holiday and school peer-support programmes (CHAMPS)	7 Goodyear et al. (2009)	This intervention was offered in two formats; as school holiday programmes run over four consecutive days or school programmes which were carried out as two-hour sessions per week over one school term or fortnightly over two school terms. It was possible to participate in both formats of the programme. The programmes aimed to provide the opportunity for children to meet regularly and offer a sense of belonging and acceptance, increase independence from parents and other adults and connectedness with peers. The programme assisted children to explore themes around the impact of hospitalization, children taking on caring responsibilities, and living with stigma.
World café	15 McAndrew et al. (2012)	This World Café event (one single occasion) had the goal of providing a platform to create important opportunities for collaboration between young carers, the volunteer sector, health and social care practice, and education. It aimed at better understanding the needs of the young people. The event was led by young service users and carers.
Psychosocial group intervention Children’s Lives Include Moments of Bravery (CLIMB®)	16 O’Neill et al. (2019)	This was a 6-week group psychosocial intervention for children, consisting of weekly 90-min sessions with art and play activities in small groups. Sessions focused on sharing one’s cancer story with other children, increasing knowledge about cancer and its treatment, normalizing and coping with feelings of sadness, anxiety and anger, as well as identifying strengths and enhancing communication with the parent with cancer. The programme was led by a professional with a health and social care background, who had completed 2 days of training in delivering the CLIMB programme.
Wonders & Worries (W&W) intervention	17 Philips & Prezio (2017)	This was a community-based psychosocial manual based intervention, designed to be individualized. It included 6 sessions (one 90-min session per week, except session 3, which was a tour of the cancer treatment centre).
Youth Education and Support (YES) pilot intervention	20 Riebschleger et al. (2009)	This was a psycho-educational support programme for youth that aimed to strengthen protective factors, help with accessing information and increase coping skills. It included six group meetings (2-h sessions) which covered different themes, such as learning, illness, effective rehabilitation, stigma, coping and hope. The sessions were led by the researcher and two professionals at two mental health agencies.
Psychosocial group intervention Children’s Lives Include Moments of Bravery (CLIMB)	21 Semple & McCaugghan (2013)	The psychosocial intervention CLIMB® was delivered at a cancer charity organization. It included 1.5 h group meetings for six consecutive weeks and were offered for children aged five to twelve years, who had a parent or a significant adult with cancer. Each week followed a similar format to help create a sense of structure and security through routine: the welcome, a team building activity, an educational component about cancer, different emotions discussed with a specific activity to help child acknowledge, express and cope with their feelings, and then a summary/closing. The goals of CLIMB® were to: [1] provide age-appropriate education about cancer, cancer treatment and the cancer experience [2]; normalize emotions that a child experiences when their parent has cancer [3]; support communication of complex emotions associated with parental cancer; and [4] improve coping by connecting children whose parents have cancer. The intervention was delivered by family-support workers.
Innovative services for children and young people	24 Templeton et al. (2011)	Three innovative programmes for children and young people living with parental substance misuse were described: Moving Parents and Children Together (M-PACT), Base Camp and Breaking the Cycle. M-PACT was a structured group programme with between three and eight families. Young people and/or their parents could join the programme. Base Camp was an intervention for children of parents with alcohol problems. A professional worker worked with each child over a period of about 6 months, offering a range of individual and group support. Breaking the Circle was an outreach service for families where the focal client was the parent with the alcohol or drug problem, but where other family members, including children were involved. A coordinator developed an individual care package and met with the family over a period of weeks or months.
Support group	25 Van Santvoort et al. (2014)	Support groups including eight weekly manual based 90-min sessions with different themes, and a booster session after 3 months. In addition, there was a meeting for parents and an individual family session. The aim of the intervention was to reduce negative cognitions; improve social support, competence, and parent–child interaction (direct intervention goals); and reduce emotional and behavioural problems (the ultimate aim of the intervention). The intervention was guided by two mental health or prevention experts (e.g. child psychologist, clinical social worker, psychiatric nurse).
Online preventive course (Kopstoring)	27 Woolderink et al. (2015).	This was an online weekly theme-based group course over 8 weeks supervised by two trained psychologists or social workers. The intervention aimed at preventing behavioural and psychological problems in children at risk.
**For the family**		
Culturally adapted family intervention (CAFI)	3 Davey et al. (2013)	This intervention included five bi-monthly support group sessions (increasing fun activities, cognitive behavioural interventions, talking about feelings/cancer, improving attachment and communication), three group meetings with children only and two family group meetings. The intervention was provided by a trained therapist.
Family-focused approach to care (theoretical model of support)	5 Foster et al. (2012)	This was a theoretical model of family-focused care. Nurses can promote parent, child, and family well-being by supporting the internal and external protective factors and reducing the risk factors. Initial identification, assessment, education, and referral, through to supportive counselling and focused family interventions are included in the model. Nurses could offer this intervention, as well as other professionals.
Family-focused psychoeducation DVD intervention based on Beardslee’s family talk intervention	8 Grove et al. (2015)	This intervention included the use of a DVD—which provided children with age-appropriate and developmentally suitable information about parental depression and anxiety, and information about how the child could respond to their parent. It also provided the children with coping and help-seeking strategies. The DVD consisted of two sections: one for parents, and one for children.
Parent-child group	11 Landry-Dattée et al. (2016)	This was a support group for children and their parents, which aimed at helping parents to communicate with their child about their disease, increasing the child’s understanding about the disease and soothing the child. The sessions were conducted by a psychoanalyst and a doctor at the hospital in the format one 2-h session every 2 weeks. The reference frame for the intervention was psychoanalytical.
A home visiting programme using a recovery planning model for families (NKC—OTCP)	13 Maybery et al. (2015)	Family recovery planning model, where the programme involved setting goals for each child and parent according to 11 predetermined domains: family connectedness, mental health knowledge, child development, education, interpersonal skills, substance abuse, lifestyle, diet and exercise, community and social connectedness, finances, family health and well-being, and accommodation. The goals formed the basis of each family member’s case management plan, short- and long-term goals were reviewed three times every 4 months. The programme was led by a case manager.
Beardslee’s family intervention (FI)	18 Pihkala et al. (2012)	The main purpose of the manualized intervention was to prevent mental health problems for children of mentally ill parents by promoting resilience in children. It included two sessions with parents focusing on their experiences and the impact of the illness on themselves and on the children. Next, individual interviews were conducted with each child, focusing on the child’s experiences of the parent’s illness and protective risk factors. A family session then took place, focusing on parent–child communication, based on the children’s questions and experiences. Follow-up was conducted after one and 6 months. The intervention was provided by professionals with training in delivering the specific family intervention.
**For the mothers**		
Mother and toddlers’ programme	23 Suchman et al. (2011)	The Mothers and Toddlers Program (MTP) was a 12-session of weekly individual parenting therapy aimed at enhancing maternal capacity for reflective functioning and softening harsh and distorted mental representations of parenting. An additional 12 weeks sessions were optional. The programme was an attachment-based individual parenting therapy for mothers involved in substance abuse treatment and caring for children from birth to 36 months. The programme was led by a MTP therapist.

Of the articles with a quantitative design, three of them described the results from randomized controlled trials [28–30] and one used a two-arm pre- and post-intervention [31]. Two observational studies also had a pre–post design [32, 33] as did one [34] of the two mixed-method articles [34, 35]. Finally, there were two cross-sectional observational studies [36, 37] while four of the included articles reported on pilot studies [28, 31–33].

### The interventions

The interventions identified in the included articles were collated and summarized according to two themes: interventions focused on the child and interventions focused on the family. The structure of the interventions focused on the child and those focused on the family had various approaches and mixed outcome effects. Seven studies were longitudinal, with more than one measurement reported. The outcome measurements varied across articles, and the outcome for the children varied, but improvements were often reported. For example; Knowledge about the parent’s illness increased in all reported studies where this aspect was measured. The children appreciated meeting with other children living in similar situations, to share experiences and engage in activities offering some fun and distraction.

#### Interventions for the child

The articles varied as to the number of participants and how the participants were grouped according to age. The articles focusing on children included participants between 2 and 19 years (Table *2*). There were seven studies which included children between 6 and 13 years, four with children between 10 to 18 years [33, 38–40] and one with children between 2 and 19 years [41]. One article reported the average age of the participating children to be 13.6 years [38].

The interventions targeting children were carried out for shorter or longer periods. Most lasted from six to 9 weeks (*n* = 10) and had modules of one-and-a-half to 2 h (*n* = 5). One article involved a school holiday programme with an optional follow-up after school programme carried out as a weekly two-hour session over one school term or fortnightly over two school terms [32]. One article did not report the duration of the intervention [38].

The various interventions for children were described in the articles as: preventive education interventions to prevent psychosocial problems, psychopathology and to help cope and ease difficult feelings related to being a next-of-kin of a parent having an illness [30, 32, 33, 42] and peer support alone or as a 4 weeks intervention program within the school holiday and/or an after school programme [32, 42]. Some were described as a psychosocial support programme [43, 44] and one as an internet café [38], while others were reported as having digitized some components [40]. One article described three new innovative services for children and young people living with parental substance abuse: first a structured group programme with a small number of families (usually between three and eight); second, offering a range of individual and group support to children over approximal 6 months, and last an outreach service for families where the client in focus is the parent with the alcohol or drug problem, but where other family members including children are involved [39]*.* Finally, one article reported about an equine-assisted therapy programme [45]*.*

#### Family interventions

In the studies that examined interventions focused on the whole family, in four of the articles, the age range of the children was between 5 and 18 years [31, 34, 46, 47]. One of the articles did not report the age of the participants [36].

Among the family interventions, the length of one intervention was not described [32], while the other four had a duration of 2 weeks to 1 year [31, 36, 46, 47]. Two of the family interventions emphasized education about how to cope with parental cancer and mental illness; one centred around family communication and children’s increased knowledge of their parents’ illness and their sense of relief by gaining a better understanding of the parents’ illness and the other about parents meeting professionals who helped them to speak about the illness and further speak with the children so they could understand it better [46, 47]. One focused on family recovery planning where a parent has a mental health or dual substance and mental health problem, making different goals for the recovery [36]. At last, one intervention was aimed only at mothers, focusing on the interaction between the mother and her infant [28].

#### Outcome measures of the interventions

The included articles reported about a range of outcome measures (Table 2). The majority of these aimed to measure burden on the child, in terms of the impact of parental illness [37], health-related quality of life [30], emotional problems [29], stress [30], anxiety [31, 41], depression [31], sleep behaviours, eating habits [41], self-concept [30], self-esteem [32], and cognition [29]. The child’s social functioning was measured in some studies, in terms of behavioural problems in the child [29], connections and relationship problems [32], and interactions with schoolmates and school performance [41]. Furthermore, some studies used coping as an outcome measure of the intervention. This concept was defined differently depending on the use of various instruments, such as coping [30], coping skills [31], adolescent coping orientation to problem experiences [33], help-seeking [34], perceived competence [29] and communication—both general communication [31] and communication about illness [41]. The child’s social support was also used as an outcome measure [29].

Furthermore, in some studies the degree of specific knowledge was measured, such as addiction-related knowledge [30], mental illness-related knowledge [34], and knowledge of psychiatric illness and recovery [33]. The child and parental relationship was another outcome measure. This was captured using instruments focusing on parent–child interaction [29], parent–adolescent relationship [31], relationship with the ill family member—specifically the ability to differentiate from parent/caregiver and to feel secure at home [41]; in one article, the relation between the child and parent was captured via structured observations of caregiving behaviour and child behaviour [28]. Children’s support needs were measured in two studies. One of these used a study-specific questionnaire concerning the types of support young people want when a parent has mental illness [29]. In the other article, an online survey was described to assess support needs for mitigating the impact of parental illness on children [37]. Finally, in three studies, the participants’ experiences of the interventions were evaluated by a satisfaction survey [31] and by completing pre-intervention and post-intervention questionnaires, around mental health knowledge, help seeking, as well as the usefulness of information provided by a DVD. There were also interviews conducted about the experiences and use of the DVD [31, 34].

### Children’s support preferences

Of the 28 articles that met the inclusion criteria in this study, 10 focused exclusively on children’s preferences for support from community health services. These articles collected children’s preferences via qualitative interviews, with the exception of two articles that used a mixed-methods approach [29] and a cross-sectional quantitative approach [37]. The support preferences described were primarily based on responses from children between 11 and 18 years of age. However, two articles included the preferences of older participants with a retrospective view [48, 49]; and two articles with participants from 11 and 26 years of age were also included, since most of the participants were within the inclusion criteria of up to 17 years of age [50, 51].

Most of the children expressed preferences for professional support (57.2–94%) [29, 31]. Children’s preferences for support are further described by themes related to their identified preferences: namely, recognition of being next-of-kin, support for the child, and support for the family.

#### Wish to be recognized as next-of-kin (*n* = 7)

Children expressed preferences for being *recognized as being next-of-kin* for an ill parent and being fully included as a relative by professionals treating or caring for their parent. This included knowing and meeting whoever was responsible for their parent’s care, e.g. the doctor [52], and being acknowledged by the responsible healthcare personnel [53]. To be recognized as a relative included having their need for information and communication concerning their parent’s illness, care and treatment acknowledged [35, 49–51]. It was important to the children to have the opportunity to talk to a competent professional who could provide them with sufficient information [35]. They also needed information that the illness was not their fault [35] and education regarding relevant legal issues [51]. However, a large proportion of children relied upon their parents for information (82.5%) [37]. Being provided information (e.g. on a DVD) in addition to that given by healthcare personnel was also preferred [35].

#### Support needs for the child (*n* = 8)

Several articles described children’s support needs around having *someone to talk* to [37, 48, 49, 51, 54]. The children needed someone to talk to about their feelings, their problems [45, 48, 51] and their parents’ diagnosis [37]. Moreover, they needed to talk to someone who could listen and understand, and who were encouraging and reassuring [49, 51]: this person could be a family member, other children participating in peer support groups and healthcare professionals. However, some needed to have some time alone, to think and manage their emotions [52].

Children expressed both a preference and need for *professional support to cope with being next-of-kin* to an ill parent. Most of the children expressed preferences for professional support (57.2–94%) [35, 37]. The children in the included articles highlighted the importance of recognizing the children’s needs and responding to these [4]. However, one article described that the children’s needs changed according to different stages of the parent’s mental illness [51], which highlights the importance of taking the illness trajectory into account when providing professional support to children. Children wished to learn how to cope with their situation and get help to make their situation manageable [29, 43]. This included help in managing their parent [48, 54], especially when newly discharged, and further access to professional help when needed [51]. Cooperation between professional support services and the children’s school was also highlighted, in that children preferred that information also was provided to the school about their parents [52]. Simultaneously, confidentiality and anonymity were important to children [35].

The children also had preferences concerning professional help regarding ways to manage the anger and emotional outbreaks they had related to their situation [37, 48, 52]. In meetings with healthcare professionals the children described that healthcare personnel at times recognised their problems without responding or not follow up by an intervention or an invitation to talk. This was perceived as an ambiguous frame and could lead to feelings of betrayal [42].

#### Support from peers, family and friends

Peer support groups were identified as another source of support for children who were an ill parent’s next-of-kin [35, 48, 52]. Children had explicit preferences for social support from peers who had been through the same experience [52, 53]. Peer support was perceived as important just after their parent’s diagnosis and during their treatment [53]. Peer support groups were considered a safe place in which the children could interact with and talk to other children in similar situations and engage in activities that provided a sense of normality. Involvement in a peer support group also fulfilled children’s preferences for distraction (respite), enabling them to have a break from their parent’s illness and difficulties at home [48, 52], have fun and relax [35, 53]. Children’s need for social support could also be met through engaging in normal life experiences, for example being with friends and their families or a neighbour [48].

#### Support needs for the family (*n* = 5)

The children expressed preferences for support, not only for themselves, but also for their family. The children reported that their family needed support to facilitate closeness and connectedness, and to facilitate better parent–child interaction while the parent was being treated in an institution [50] and in general [54]. The children also reported a need to spend time with their ill parent and play an active role in the family [52]. Here, family support included both financial support [48] and practical support [48].

## Discussion

### Summarizing the main results

The current scoping review aimed to identify: 1) current knowledge about community-based support for children 0 to 17 years of age who are next-of-kin living with a parent experiencing illness or disability, and 2) children’s preferences and expressed needs for support. The results indicate that there are existing supportive interventions for children who are next-of-kin for parents experiencing physical and mental illness developed in communities. Most interventions identified were related to mental illness, including substance abuse. No article was identified involving supportive interventions in relation to living with a parent with disability.

The interventions were offered to children of all ages, but more often to children in the higher age-span. The interventions typically had a duration of two to 3 months. Moreover, although most interventions did show some effect after a short period, no intervention was evaluated after 6 months. There were 19 interventions, 12 had a child focus, 6 family focus and one a mother and child focus. The child focused interventions largely centred on giving the children additional knowledge about the illness, helping them communicate with their parents about difficult challenges, such as shame, living in a family with an ill parent; and alleviating guilt they might be feeling about living their own lives. Of the articles aimed at children’s preferences or need for support, children aged 11 to 18 years were primarily the ones who voiced their experiences. They revealed a need for support for themselves, as well as for the entire family; moreover, they wanted to be recognized as a next of kin such as a grown up, despite being a minor. There is a limitation that support preferences and/or needs of children 10 years and under were not reported in the qualitative articles identified.

### Methodological considerations

The primary strength of the scoping review as a research method is that it enables the inclusion of articles with a range of methodologies; as such, the reviews can draw on and aggregate a broader knowledge base and more comprehensively address gaps in the literature [23]. The present study comprises papers reporting on both qualitative and quantitative designs, and one theoretical paper.

The validity and reliability of this study was strengthened through adherence to the stages of performing a scoping review as recommended by Levac and colleagues [23] and to Tricco et al. [21] reporting guidelines. The use of PICO resulted in explicit eligibility criteria that guided the searches and selection process, as well as each stage of the review process. Another strength was that the research team included researchers with competence in the field and the methods used. The research team also included an academic librarian trained in performing searches for systematic reviews. This ensured a transparent search process, for which the full searches are visible at https://hdl.handle.net/11250/2688211. The searches included eight relevant databases for the topic of interest using both subject terms and free text search terms, and searches in reference lists that resulted in additional references. This contributed to both breadth and depth in the searches, which is important for a comprehensive scoping review of the existing literature [23]. However, a limitation is that only research articles and theoretical papers were included (and thus not e.g. grey papers), due to the timeframe of this project.

The team of researchers worked closely together to ensure rigour at each stage of the review. For example, the selection of the articles and data extraction were first performed independently by the researcher and then discussed in pairs. Uncertainties and disagreements were discussed by the researchers before a decision was taken about inclusion or exclusion and about what data should be extracted from the articles. Similarly, the collating and summarizing of the data were discussed until consensus was reached. This increased the validity and reliability of the selection and the interpretation process [23].

The inclusion criteria of this study specified including children from 0 to 17 years of age. However, the articles sometimes included children above 17, or included older children who talked about their experiences retrospectively. We chose to include these articles because these still contributed important knowledge for the field.

Articles were included if they had provided substantial findings from the children’s perspectives. However, many articles were excluded when the children’s perspectives, preferences or outcome measures were not the focus or were clearly separated from other perspectives (e.g. that of the parents or healthcare provider). This was often seen in articles about support for new-borns, and therefore few articles were included in the review for this age group. Further research on children’s support preferences should therefore clearly separate and highlight the findings regarding children’s preferences, specifically.

Of the articles included, 17 were reports regarding interventions to children. These had two different areas of focus: supporting the child and supporting the family, including the mother. However, each article that was included in the review typically evaluated one specific intervention. Several evaluations of the same type of intervention in different contexts would strengthen the validity of the intervention. Additionally, the evaluation of the interventions was performed using a wide range of different outcome measures, hindering accurate comparison of the effect of the interventions.

Developing interventions is complex. According to Craig et al. [55] framework, the process of developing interventions should include describing the development process (identifying the evidence base), piloting and feasibility testing, evaluating (assessing effect) and implementing the intervention (disseminating, long-term follow up). None of the interventions identified in this review were found to fulfil these criteria/stages. As such, more research is needed before recommendations regarding further implementation can be made about the interventions presented.

The themes representing the children’s preferences for support were largely based on articles from the western world (e.g. Europe, the United States and Australia), and one article from Iran. This highlights the need for more studies from other parts of the world. However, the articles that were included in this review were from several different countries, involving children with different ages, genders, and parents experiencing a variety of illnesses. The preferences for support seem to have more similarities than differences regarding countries, culture and parental illness. This should contribute to the transferability of the findings to various contexts.

Despite the comprehensive literature search conducted for this study, this review is unable to give a complete picture of children’s preferences for support and which interventions proved most useful for children as next-of-kin to a parent having an illness or disability. However, this review has identified an urgent need to identify these children’s needs and preferences for support and further develop community-based supportive interventions.

## Conclusion

This review has presented supportive interventions from community health and social services for children of different ages who are next-of-kin to a parent who experiences illness or disability and has highlighted the importance that these children should receive support according to their preferences. This insight, informed by the children’s own voices, is essential if community-based services are to be developed for and linked to this population. If their needs are not met, they are at risk developing mental, social and behavioural problems, which might impact education, social opportunities and employment prospects.

### Relevance to clinical practice

The results indicate that children want to be recognized as next-of-kin. According to the *Convention on the rights of the child* [6] children are recognized as vulnerable with a special need for protection and support, but they have the right to be given information and involved in decisions that concern them [6]. However, previous research has identified that parents and professionals are reluctant to talk with children about parental illness, due to a lack of knowledge around what to say, a fear of upsetting the child or making things worse, and the belief that the child is not mature enough to understand [56]. Children who grow up with parents experiencing a mental or somatic illness or disability experience a range of concerns—including conflict-filled family interactions, poorer parental functioning and neglect—and are at higher risk of developing mental, social and behavioural problems [57, 58]. These risks may be mitigated if children in these challenging situations are offered support, predictability and thus security. As results from the current review show, there are a variety of supportive interventions that could be offered within the community, which could benefit children as next-of-kin and which are in line with their expressed needs and preferences.

As noted earlier, community nurses work extensively with children and their families around the world [15] and are therefore well-positioned to do this work. In Norway, the PHNs meet a substantial number of children through their work in the health centres and school health services, where they have the responsibility to promote health among children [16, 17]. The PHNs play an important role in discovering early development of health problems among young people and can initiate necessary referrals to other authorities. As such, PHNs should be aware of the children who are their parents’ next-of-kin and have a duty to protect these children. Meeting children’s special needs via supportive interventions, in line with those identified in the current study, may be regarded as an important and appropriate measure in health promotion and illness prevention. According to the International Council of Nurses’ framework [59], a nurse acts in an advocacy role to protect human rights and questions violations of client in accordance with jurisdictional and the ICN. Even though nurses work to change policies, and health care professionals are expected to support children, they do need training and expertise to do this in an evidence-based way, to not cause any harm.

The articles in this review described interventions that dealt with knowledge provision, designed as education programmes for the child and/or the whole family. In both the child- and the family-centred interventions, children primarily wanted knowledge—specifically, knowledge about the parent’s illness—as well as communication, recovery planning and predictability. PHNs are effective information and education resources for patients who need assistance around coping with changes in health. Indeed, their professional responsibilities involve recognizing opportunities, providing guidance and educating individuals, families and communities—moreover, they have the ability to tailor teaching and learning strategies to the needs and characteristics of these children and their families [59].

To summarize; first all children as next of kin have the right to be identified, not only those whose parent is treated within specialized health-care. Thus, national legislations need to be adapted accordingly, suggesting which authority should have the responsebility for the children as next of kin. Secondly, the authorities should provide the appointed professionals with relevant guidelines, knowledge and methods needed for approaching children with adequate questions to identify those in need of support. The PHN working in different arenas in the community, such as at health service centres or schools could be one central profession whose role is it to identify children in need for support, for providing some aspects of support, or referring the child and parents for further support. Depending on whether a child is caring about a relative or caring for a relative [3] the need for support will vary, and if more complex needs are identified, interprefessional collabortation within and between authorities might be needed. Supportive interventions should preferable be evidence-based and they need to be evaluated, both on individual and group level.

### Identification of research gap

In this scoping review, no articles were identified that involved supportive interventions in relation to living with a parent with a disability. The support preferences and needs of children up to 10 years of age were not reported in the articles that focused on preferences and needs; moreover, the structure of the interventions for children and for families had different approaches and mixed effects, depending on the outcome measures used. Future studies should focus on 1) how to identify these children in the community, 2) children who have a parent with a physical or psychological disability, and 3) the youngest children. Moreover, more studies are needed which use instruments and methods that are comparable, as well as long-term interventions and follow-up/evaluation studies.

## Data Availability

All data generated or analyzed during this study are included in the article. The earch strategy s is available from https://hdl.handle.net/11250/2688211

## Abbreviations

PHN: Public Health Nurse
PRISMA: Preferred Reporting Items for Systematic Reviews and Meta-Analyses
IMRAD: Introduction - Method – Results – and – Discussion
